# Assessment of fuzzy logic to enhance species distribution modelling of two cryptic wood boring beetle species in Australia

**DOI:** 10.1038/s41598-024-77533-0

**Published:** 2024-11-13

**Authors:** Xingyu Li, Robert N. Emery, Grey T. Coupland, Yonglin Ren, Simon J. McKirdy

**Affiliations:** 1 Centre for Biosecurity and One Health, Harry Butler Institute, Murdoch University, Murdoch, WA 6150 Australia; 2 School of Science, Health, Engineering and Education, Murdoch University, Murdoch, WA 6150 Australia

**Keywords:** Fuzzy logic, Species distribution modelling, Habitat suitability, Environmental favourability, Fuzzy similarity, Fuzzy intersection, Environmental impact, Computational biology and bioinformatics, Environmental sciences, Ecological modelling

## Abstract

Fuzzy logic presents a promising approach for Species Distribution Modelling by generating a value that can be used for comparative purposes termed ‘environmental favourability’. In contrast to ‘presence probability’, ‘environmental favourability’ remains robust regardless of species prevalence. This characteristic facilitates effective comparisons across species with varying levels of prevalence. In this study, presence probability was predicted using three commonly used Species Distribution Models: Generalised Linear Model, Generalised Additive Modelling, and Boosted Regression Trees for two beetle species, *Euwallacea fornicatus* and *Euwallacea perbrevis* in Australia. Fuzzy logic was then employed to derive environmental favourability values based on these models. Additionally, Maxent modelling was included to compare prediction outputs and facilitate a comprehensive analysis. Model performance was evaluated using standard metrics (Area under the receiver operating characteristic curve, True statistical skill, Correct classification rate), as well as Hosmer-Lemeshow test. The research explored fuzzy similarity, fuzzy intersection and potential biotic interaction of these closely related borers, and revealed a favourable distribution pattern for *Euwallacea fornicatus* across Australia. This study supports the efficacy of fuzzy logic in Species Distribution Modelling and highlights the value of environmental favourability function in enhancing the comparative analysis of the geographical relationship across species. This approach offers a more nuanced perspective on Species Distribution Modelling.

**Supplementary Information:**

The online version contains supplementary material available at 10.1038/s41598-024-77533-0.

## Introduction

As a widely used tool in ecology and biosecurity, Species Distribution Models (SDMs) are generally restricted by the need for presence/absence data along with the response of species to a set of predictors^1^. Specifically, the prevalence (proportion of species presences) is an unavoidable restriction that affects SDM predicted distribution areas^2,3^. These impacts refer to the important range of distributed probability^4^. In other words, the distributed probability for restricted species may be underestimated, while more widely spread species could be overestimated. Hence, the comparison of distributed probability between different species that have varying prevalence, is difficult in practice.

To provide a comparative output between different species, fuzzy set theory^5^ introduced the roles of fuzzy logic to provide a realistic continuum degree of membership to classify the data^3,6^. Specifically, fuzzy logic resolved the issues presented by small-range species for which minute differences in occurrence data may indicate whether or not they coincide substantially in their distribution ranges^7^. With regard to the theoretical approaches, the proposed formula (1) for the *probability* output of logistic regression is the following:

1 $$\:\varvec{P}=\frac{{\mathbf{e}}^{\varvec{\alpha\:}+{\varvec{\beta\:}}_{1}{\varvec{x}}_{1}+{\varvec{\beta\:}}_{2}{\varvec{x}}_{2}+\cdots\:+{\varvec{\beta\:}}_{\varvec{n}}{\varvec{x}}_{\varvec{n}}}}{1+{\mathbf{e}}^{\varvec{\alpha\:}+{\varvec{\beta\:}}_{1}{\varvec{x}}_{1}+{\varvec{\beta\:}}_{2}{\varvec{x}}_{2}+\cdots\:+{\varvec{\beta\:}}_{\varvec{n}}{\varvec{x}}_{\varvec{n}}}}$$

Where *P* is the *probability of presence* of a species, *e* is the basis of the natural logarithm, and *α* is a constant and *β*_*1*_, *β*_*2*_, *.*,* β*_*n*_ are the coefficients of the *n* predictor variables *x*_*1*_, *x*_*2*_, *.*,* x*_*n*_^8,9^.

Based on the roles of fuzzy logic, Real, et al.^9^ introduced a mathematically-based environmental favourability function, built with binomial distribution and logit link functions. This function provides an *environmental favourability* value, which represents a fuzzy membership indicating *possibility* rather than *probability*^9,10^. The proposed formula for *environmental favourability* is the following:

2 $$\:\varvec{F}=\frac{{\mathbf{e}}^{{\varvec{\alpha\:}}_{1}+{\varvec{\beta\:}}_{1}{\varvec{x}}_{1}+{\varvec{\beta\:}}_{2}{\varvec{x}}_{2}+\cdots\:+{\varvec{\beta\:}}_{\varvec{n}}{\varvec{x}}_{\varvec{n}}}}{1+{\mathbf{e}}^{{\varvec{\alpha\:}}_{1}+{\varvec{\beta\:}}_{1}{\varvec{x}}_{1}+{\varvec{\beta\:}}_{2}{\varvec{x}}_{2}+\cdots\:+{\varvec{\beta\:}}_{\varvec{n}}{\varvec{x}}_{\varvec{n}}}}$$

Where *F* is the *environmental favourability* value, and *α*_*1*_ is the parameter that is estimated iteratively based on the values of the predictor variables^9^.

The degree of membership reflects how likely pixels fall within the set of potential species occurrence areas^7^. Fuzzy set having degrees of membership from 0 to 1. Comparing the relevance of *probability* and overall *prevalence*, the *environmental favourability* values reflect the biogeographical relationship between the species and the predictors, independent from known species *prevalence*^9,11,12^.

A symbiotic mutualist organism can make the associated pest more complex and increase the risk to biosecurity. For example, the *Euwallacea* spp. species complex Eichhoff *sensu lato* (Coleoptera: Curculionidae: Scolytinae: Xyleborini) is a tribe of wood-boring, fungus-farming ambrosia beetles with a complex lineage and genetic divergence^13^. These beetles are known to attack a wide range of hosts, over 400 species, and have established around the world^13–15^.

The clades of *Euwallacea* spp. associated with their mutualist Ambrosia Fusarium Clade (AFC) are responsible for damage to tree hosts^16^ including several economically important crops including avocados and tea plants^17–19^. The fungal symbionts play a role in survival of *Euwallacea* spp. and proliferation^20,21^. Furthermore, the fungal symbionts invade the trees vascular tissues, causing branch dieback which often destroys the tree^18,22^. In addition, O’Donnell, et al.^17^ reported that some pest genera of *Euwallacea* spp. are capable of carrying one or more fungi, which are able to share and switch symbionts. This new symbiotic form may make the fungi more pathogenic if hybridized with exotic strains^23^.

Currently there are two clades from this cryptic species complex in Australia, located thousands of kilometres apart. One is *Euwallacea fornicatus*, Polyphagous shot hole borer (PSHB), which was detected in East Fremantle (Latitude/Longitude: -32.03823° / 115.76759°), Western Australia (WA), on 6 August 2021 via the citizen science app, MyPestGuide Reporter™ (https://www.agric.wa.gov.au/pests-weeds-diseases/mypestguide)^24,25^. The other is *Euwallacea perbrevis*, Tea shot hole borer (TSHB), which has been identified as being in Australia for many years, and possibly part of a native range^26^. The first clear record of *E. perbrevis* was on the Sunshine Coast (Latitude/Longitude: -26.50001° / 152.99999°), Queensland (Qld) in 2009, followed by the Atherton Tablelands (Qld) in 2011^27^ and afterwards in northern New South Wales (NSW). *Euwallacea perbrevis* was detected occasionally during surveys for another ambrosia beetle *Xyleborus glabratus* (Eichhoff 1877) in 2010^28^–^30^. *Euwallacea perbrevis* is not originally native to Australia, but it could be regarded as naturalised due to its prolonged establishment in Qld^31^. Interestingly, *E. perbrevis* has been present on the east coast of Australia for many years but it has not been recorded in other states not contiguous with Qld, including WA, where its close relative *E. fornicatus* was recently reported.

*Euwallacea* spp, with low dispersal propensity can only spread short distances independently^28^. However, the borers can be spread long distance through transportation of plants, nursery stock, and green waste from urban areas. Additionally, there is evidence suggesting that *E. fornicatus* can travel significant distances under favourable conditions, such as strong winds^28^. Notably, the borer has been reported to have spread to Rottnest Island, 20 km off the coast of WA^32^, possibly as a result of wind dispersal. Furthermore, *E. fornicatus* also have congenital advantages that permit easy concealment, they have a polyphagous nature and remain inside the tree farming fungus^33^, all of which assist establishment and colonization^34^.

The unexpected recent arrival of *E. fornicatus* on the west coast of Australia and the long-term presence of *E. perbrevis* on the east coast could impact inter-state wood movement, potentially creating biosecurity concerns. The complex and ambiguous taxonomic history of *E. fornicatus* and *E. perbrevis*, has led to confusion regarding the economic importance of these two species^35^. It is economically and environmentally important to model the favourable areas for these two borers across Australia to assess their relationship and potential spread.

Fuzzy set theory has demonstrated the value of environmental favourability in SDMs, with successful applications documented in many studies^2,9,36^. Real, et al.^9^ was an early adopter of applying an environmental favourability function based on a fuzzy logic framework, which notably enhanced the comparative SDMs for the *Galemys pyrenaicus* Pyrenean desman in Spain. Similarly, Acevedo, et al.^36^ utilized environmental favourability SDM to assess three hare species *Lepus* spp. in Europe and their response to climate change, effectively highlighting the competitive advantages of one species in the environment and identifying potential threats to species coexistence posed by climate change. Additionally, Barbosa and Real^2^ demonstrated the utility of environmental favourability functions in directly comparing predictions for two toad species *Bufonidae*, which significantly emphasized the benefit of fuzzy logic in integrating multi-species models for conservation planning.

However, the concept of fuzzy logic and application of environmental favourability function have yet to be widely integrated or applied across a broad range of species, especially those impacting biosecurity. In this study, fuzzy logic is employed to derive environmental favourability values based on the probability of presence obtained from SDMs. This approach enables direct comparison of predictions for *E. fornicatus* and *E. perbrevis* without the confounding effects of differing prevalences between the two borers. Additionally, the use of environmental favourability facilitates the analysis of biotic interactions and biogeographical relationships between the two species for further analysis. This paper proposes environmental favourability analysis based on fuzzy logic as a tool to support improvement of different SDMs using two exemplar species – *E. fornicatus* and *E. perbrevis*. The aims of this study are:

(i) to demonstrate that incorporating environmental favourability can enhance Species Distribution Models by providing more detailed and informative results through comparison of two borer species *E. fornicatus* and *E. perbrevis* in Australia.
(ii) to compare the intersection areas of environmental favourability and analyse the geographical relationship for exemplar species *E. fornicatus* and *E. perbrevis*.

## Materials and methods

### Species occurrence data

**Fig. 1:**
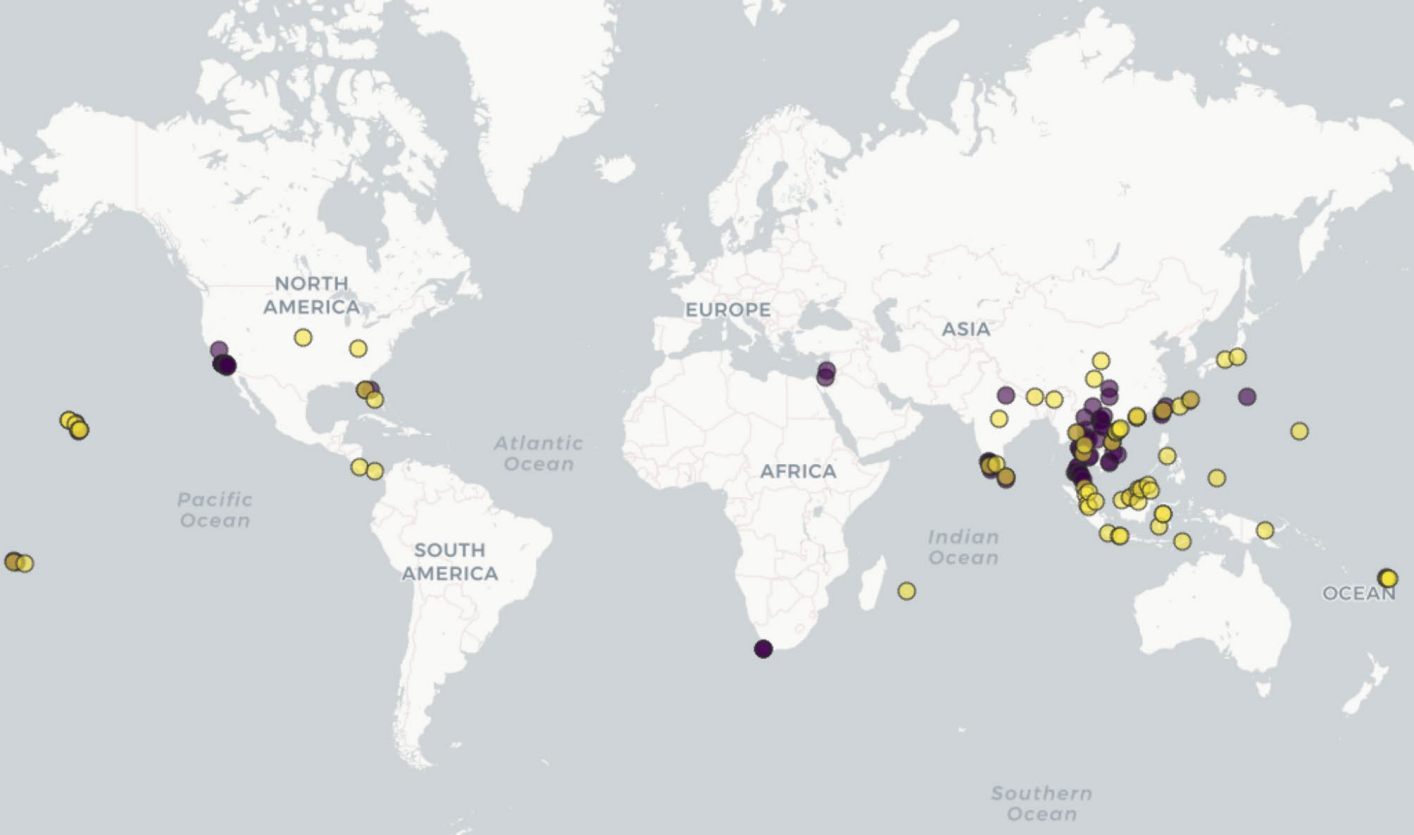
Global occurrence data (exclusive of Australian records) of *Euwallacea fornicatus* (purple circle) and *Euwallacea perbrevis* (yellow circle) used for modelling. The species global occurrence map was conducted using R Studio (Version 4.4.0) (http://www.rstudio.com/). Global occurrence data of *Euwallacea fornicatus* were obtained from Global Biodiversity Information Facility (GBIF; https://www.gbif.org/), Centre for Agriculture and Bioscience International (CABI; https://www.cabi.org/cpc), and European and Mediterranean Plant Protection Organization (EPPO; https://www.eppo.int/) and from published literature^14,37^.

The occurrence data of *Euwallacea fornicatus* and *Euwallacea perbrevis* were collected from currently available distribution records across a range of sources: Global Biodiversity Information Facility (GBIF; https://www.gbif.org/); Centre for Agriculture and Bioscience International (CABI; https://www.cabi.org/cpc); European and Mediterranean Plant Protection Organization (EPPO; https://www.eppo.int/) as well as recent occurrence data ascertained from literature with distribution data^14,37^. Sampling bias can result in spatial autocorrelation and associated overestimation of model performance. This can obstruct model application and interpretation^38–41^. To alleviate this problem, this study used the ‘spThin’ package version 0.2.0^42^ in R Studio (Version 4.4.0) (http://www.rstudio.com/) to reduce the species occurrence records in geographical space. In total, the modelling worked with 89 *E. fornicatus* and 66 *E. perbrevis* records (Fig. 1).

## Environmental data

Nineteen bioclimatic variables at 10 arc-minutes spatial resolution between 1970 and 2000 were downloaded from the WorldClim dataset^43^, created using climate interpolation methods originally developed for the Bioclim database^44^. Pearson’s correlation coefficient^45^ was introduced to reduce multicollinearity caused by a close relationship between one variable and a set of other variables^46^. The variables with higher pairwise correlation coefficients (| r | >0.8) were removed leaving seven variables for modelling (Table 1).

**Table 1:** Bioclimatic variables from the WorldClim used for modelling the potential establishment of *Euwallacea fornicatus* and *Euwallacea Perbrevis* across Australia.

Code	Variable title	Unit	Variables used for modelling
Bio1	Annual mean temperature	°C	
Bio2	Mean diurnal range	°C	$\:\surd\:$
Bio3	Isothermality	%	
Bio4	Temperature seasonality	°C	
Bio5	Maximum temperature of warmest month	°C	
Bio6	Minimum temperature of coldest month	°C	
Bio7	Temperature annual range	°C	$\:\surd\:$
Bio8	Mean temperature of wettest quarter	°C	$\:\surd\:$
Bio9	Mean temperature of driest quarter	°C	$\:\surd\:$
Bio10	Mean temperature of warmest quarter	°C	
Bio11	Mean temperature of coldest quarter	°C	$\:\surd\:$
Bio12	Annual precipitation	mm	$\:\surd\:$
Bio13	Precipitation of wettest month	mm	
Bio14	Precipitation of driest month	mm	
Bio15	Precipitation seasonality	%	
Bio16	Precipitation of wettest quarter	mm	
Bio17	Precipitation of driest quarter	mm	$\:\surd\:$
Bio18	Precipitation of warmest quarter	mm	
Bio19	Precipitation of coldest quarter	mm	

## Species distribution models development

In this study, four commonly used Species Distribution Models (SDMs) were employed for initial model development: Generalised Linear Model (GLM), Generalised Additive Modelling (GAM), Boosted Regression Trees (BRT) and Maximum Entropy Modelling (Maxent). The outputs of the initial modelling comprising probability values were generated using ‘terra’ package^47^ with R Studio (Version 4.4.0) (http://www.rstudio.com/). A total of 10,000 random background points were created as pseudo-absence points. In this study, ‘cross-validation’^41^ with five-folds and ten replications was employed to mitigate overfitting associated with pseudo-absence data^48^ and to reduce sampling bias that could impact model performance^49^. GLM, GAM, and BRT provided predictions in terms of probability of presence, while Maxent produced a more interpretable ‘cumulative’ representation, which reflects relative habitat suitability rather than direct estimates of probability of presence^50,51^. Recent studies have shown that Maxent is equivalent to a Point Process Model (PPM) in large samples^52,53^. This indicates that Maxent can be effectively utilized to fit PPMs and can help identify and address certain types of sampling bias^54^. Therefore, it is worthwhile to use Maxent outputs for a comprehensive comparison. Subsequently, all prediction maps were visualized using ‘ggplot2’ package^55^ in R for further analysis and interpretation.

## Fuzzy logic analysis and environmental favourability

Building upon the outcomes of previous SDMs, fuzzy logic analyses were conducted using R Studio (Version 4.4.0) (http://www.rstudio.com/) with ‘fuzzySim’ package (Version 4.3)^7,9^ and ‘modEvA package’ Version 3.5^56^. The environmental favourability values were initially derived used ‘Fav’ function from ‘fuzzySim’ package based on presence probability outputs from previously employed SDMs including GLM, GAM and BRT. Environmental favourability values ranging from 0 to 1 indicate the degree of membership on each site which correspond to how favourable the environment is for the species^9,11^. Environmental *favourability* is obtained directly from *probability* data, which offsets the uneven proportions of presences and absences among the occurrence data^9^. Therefore, Maxent outputs, which provide relative habitat suitability rather than direct estimates of environmental favourability, were not converted into favourability values^57^ as these relative suitability values do not directly correspond to environmental favourability. Thus, Maxent’s relative habitat suitability values were used for comparative analysis with the environmental favourability predictions.

Subsequently, to provide a biogeographical analysis between *E. fornicatus* and *E. perbrevis*, this study assessed the environmental favourability intersection values. The fuzzy intersection was calculated using ‘sharedFav’ function from ‘fuzzySim’ package with a confidence level of 0.95. This output represents the intersection of environmental favourability values for *E. fornicatus* and *E. perbrevis* at each pixel within Australia. Fuzzy intersection is able to provide simultaneous environmental favourability for the two borer species^9^. The degree of membership for each cell within the intersection area is determined by the minimum environmental favourability values for *E. fornicatus* and *E. perbrevis* in the cell^11^. Environmental favourability intersections were visualized using ‘ggplot2’ function for graphical presentation. Additionally, the similarity index is widely used in ecology for detecting species distributional associations^58^. Fuzzy similarity indices are preferred for defining distributional relationships due to their robustness against disparities, errors or gaps in occurrence data^7^. Pair-wise fuzzy similarity of the distributional relationship between *E. fornicatus* and *E. perbrevis* was computed using the Jaccard method^59^ with ‘simMat’ function from ‘fuzzySim’ package.

Additionally, the environmental favourability values for *E. fornicatus* and *E. perbrevis* were further used to compute a numeric value – Fuzzy overlap index (FOvI). This index quantifies the overall similarity in environmental requirements between the two borer species across Australia^60^. The FOvI estimates the degree to which environmental conditions in Australia are simultaneously favourable for both *E. fornicatus* and *E. perbrevis*. The value ranges from 0 to 1 represent no distributional overlap (0) to identical distribution (1)^7,60^. To further analyse the trends in environmental favourability for both species, this study illustrated the biogeographical relationship between *E. fornicatus* and *E. perbrevis* using a shared environmental favourability plot. This plot segmented the FOvI values into ten intervals with the curve shape highlighting the balance of environmental favourability between the studied species^60^.

## Model evaluation and validation

Model evaluation was conducted using ‘modEvA’ package within R Studio (Version 4.4.0) (http://www.rstudio.com/). Five commonly used evaluation metrics were employed to assess the model performance: Area Under Curve (AUC), True Statistical Skill (TSS), Correct Classification Rate (CCR), Sensitivity and Specificity. The Area Under the Receiver Operating Characteristic (ROC) Curve (AUC)^61^ was used to evaluate the discrimination capacity. The AUC value ranged from 0 to 1, with 0.5 indicating performance worse than a random model, with performance increasing as the value approaches 1 ^62^. The evaluation metrics also included TSS^63^ that is independent from prevalence. TSS values range from − 1 to + 1, representing performance no better than random (-1) to perfect agreement (+ 1)^63^. CCR was used to measure overall accuracy^61^ and it ranges from 0 to 1 indicating the model adequacy^61,64^. Additionally, sensitivity and specificity are critical factors for assessing ideal model performance in ecological modelling^63^. Sensitivity and Specificity measure omission and commission errors, respectively. Sensitivity refers to the proportion of correctly predicted suitable areas among observed presences, while Specificity denotes the proportion of correctly predicted unsuitable areas among observed absences^63,65,66^. Furthermore, these metrics assess robustness to prevalence, indicating the proportion of background sites where the species was recorded^63^.

This paper also introduced Hosmer-Lemeshow goodness of fit (HL test)^67^ to assess the calibration performance and reliability of the models in terms of decile bins, which divided the probability^68^ using ‘HLfit’ function from ‘modEvA’ package. In essence, HL test measures how well a model fits the observed data. It is a statistical test typically providing three key metrics including chi-squared (chi.sq), p-value (P.value), and Root Mean Squared Error (RMSE)^67^. The chi-squared is a metric that accesses the discrepancy between observed and expected frequencies, with a lower chi.sq value indicating a better model fit^67^. P-value evaluates the statistical significance of the chi-squared statistic, where higher P-values suggest a better fit^69^, while lower P-value indicate a lack-of-fit^68^. Additionally, HL test computes the root mean squared error (RMSE), which quantifies the average magnitude of prediction errors. RMSE measures the absolute fit of models to the data with values orienting from 0 to ∞ perfect fit to poor fit^67,68^.

To provide a complementary and informative characteristic of the predictive performance of models, fuzzy entropy^70^, applied from the environmental favourability values, was introduced to assess uncertainty. Estrada and Real^71^ identified fuzzy entropy as an indicator of the uncertainty of a system and with the application of fuzzy set, the fuzzy entropy is no longer zero since all the values are fuzzy. The fuzzy entropy value yields a continual degree of membership, with a range from 0 to 1 rather than strict 0 or 1^72^. The fuzzier the value, the higher fuzzy entropy, indicating more uncertainty as the species distributions are gradually constrained by the environment^71^.

Furthermore, the occurrence records of *E. fornicatus* and *E. perbrevis* in Australia were utilized to validate the predictions. These occurrence records were overlaid on all the predicted outcomes using ‘ggplot2’ package within R Studio (Version 4.4.0) (http://www.rstudio.com/).

## Results

### Prediction maps for probability, favourability and suitability

**Fig. 2:**
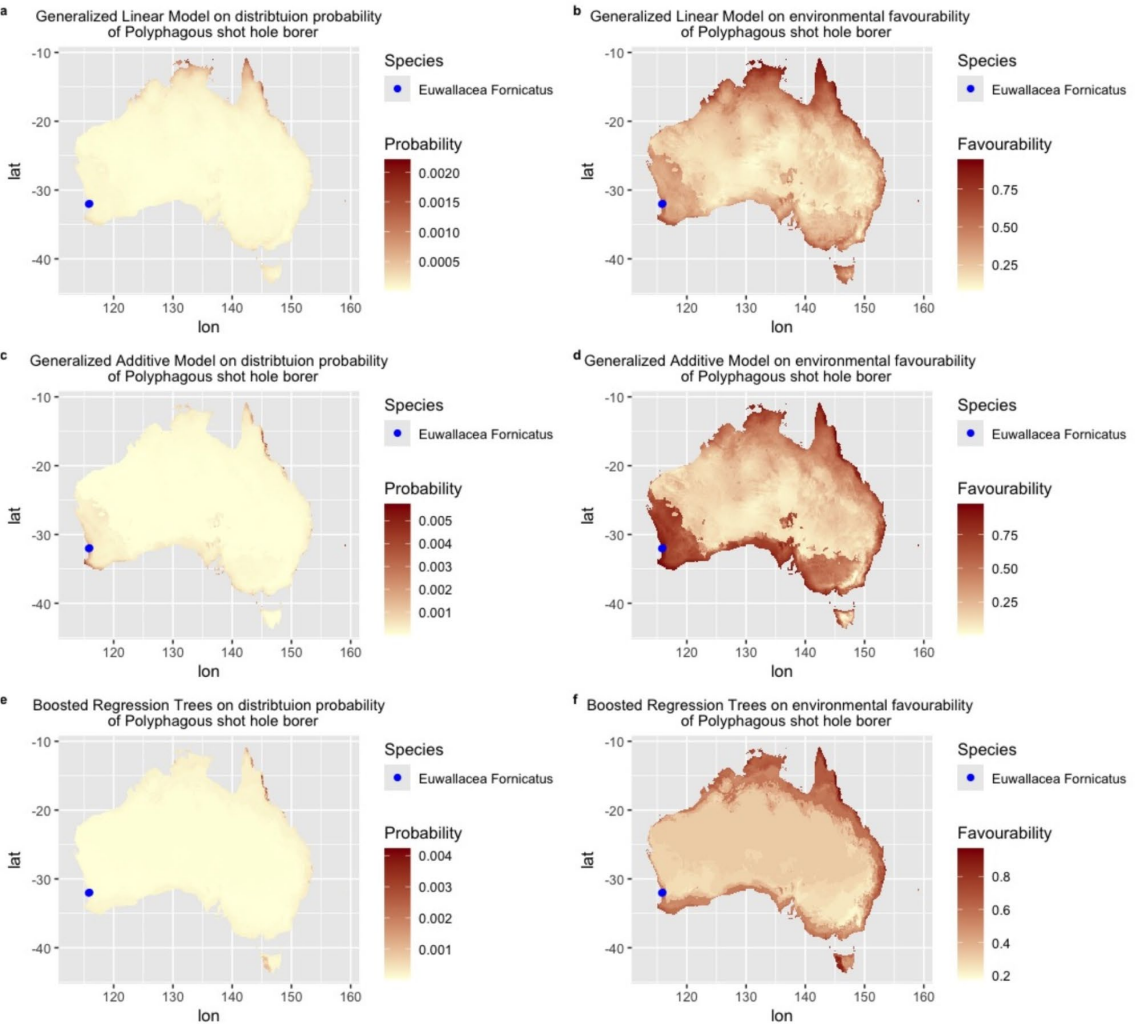
Prediction maps of *Euwallacea fornicatus* across Australia with multiple Species Distribution Models: Generalized Linear Model: (**a**) probability of presence, (**b**) environmental favourability; Generalized Additive Model: (**c**) probability of presence, (**d**) environmental favourability; Boosted Regression Model: (**e**) probability of presence, (**f**) environmental favourability. The blue dot indicates occurrence records of *Euwallacea fornicatus* in Western Australia. Global bioclimate data were acquired from the WorldClim open database (https://worldclim.org). The Species Distribution Model and Favourability Function was conducted using R Studio (Version 4.4.0) (http://www.rstudio.com/). Occurrence records of *Euwallacea fornicatus* in Australia were obtained from Global Biodiversity Information Facility (GBIF; https://www.gbif.org/).

**Fig. 3:**
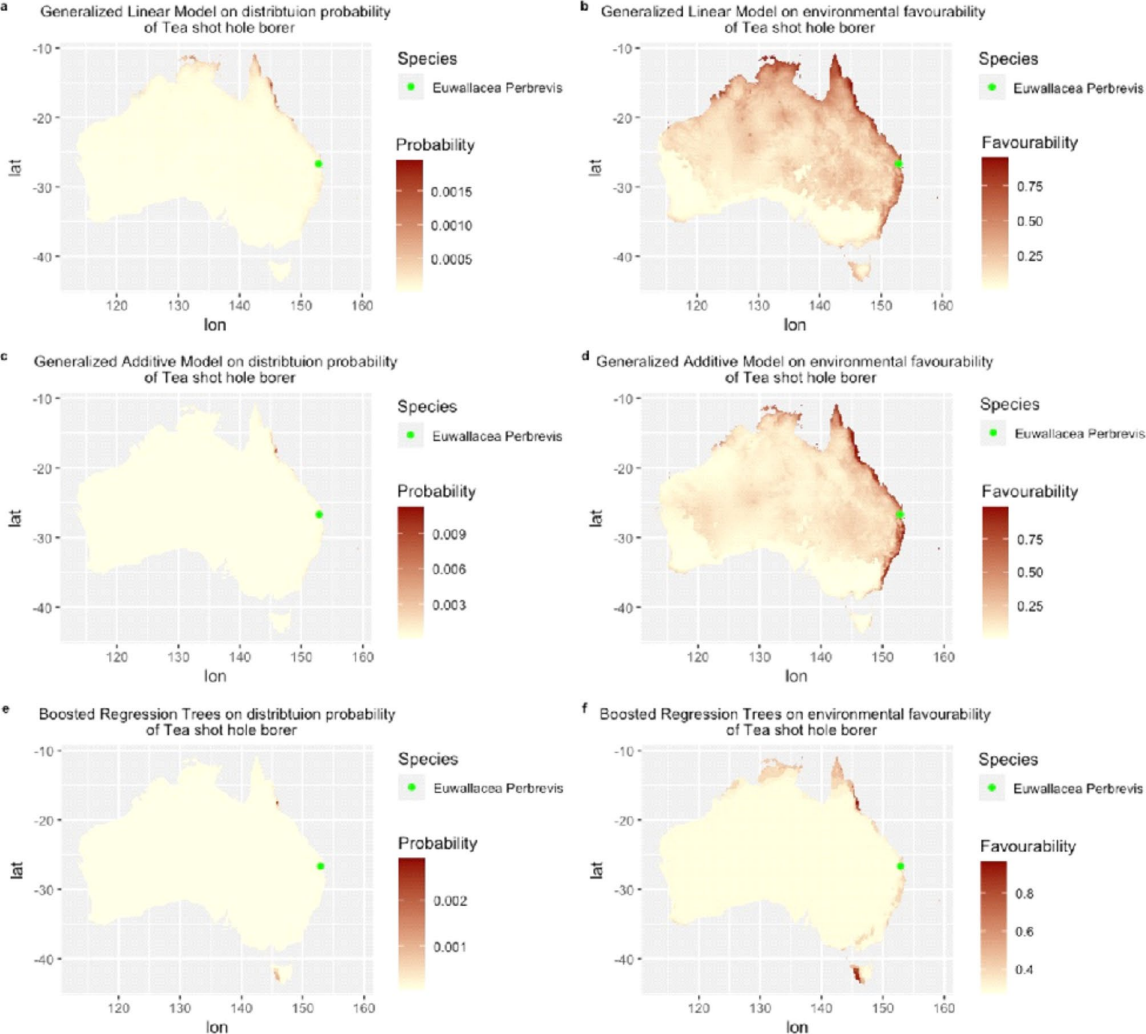
Prediction maps of *Euwallacea perbrevis* across Australia with multiple Species Distribution Models: Generalized Linear Model: (**a**) probability of presence, (**b**) environmental favourability; Generalized Additive Model: (**c**) probability of presence, (**d**) environmental favourability; Boosted Regression Model: (**e**) probability of presence, (**f**) environmental favourability. The green dot indicates occurrence records of *Euwallacea perbrevis* in Queensland. Global bioclimate data were acquired from the WorldClim open database (https://worldclim.org). The Species Distribution Model and Favourability Function was conducted using R Studio (Version 4.4.0) (http://www.rstudio.com/). Occurrence records of *Euwallacea perbrevis* in Australia were obtained from Global Biodiversity Information Facility (GBIF; https://www.gbif.org/).

The prediction maps for *Euwallacea fornicatus* illustrate presence probability and environmental favourability derived from Generalised Linear Model (GLM) (Fig. 2a, b), Generalised Additive Modelling (GAM) (Fig. 2c, d), and Boosted Regression Trees (BRT) (Fig. 2e, f). The corresponding prediction maps for *Euwallacea. perbrevis* presented in Fig. 3 follow the same order. All occurrence records for *E. fornicatus* and *E. perbrevis* are located within the predicted areas of environmental favourability as indicated by all models.

The Species Distribution Models (SDMs) for both *E. fornicatus* and *E. perbrevis* produced significantly varying maps of presence probability and environmental favourability across Australia (Figs. 2 and 3). The probability of presence maps (Figs. 2a, c and e and 3a, c and e) generated by three SDMs showed relatively lower predicted areas for both species compared to the fuzzy prediction maps (Figs. 2b, d and f and 3b, d and f) derived from environmental favourability modelling. The latter maps exhibited a more extensive predicted range for both species.

**Fig. 4:**
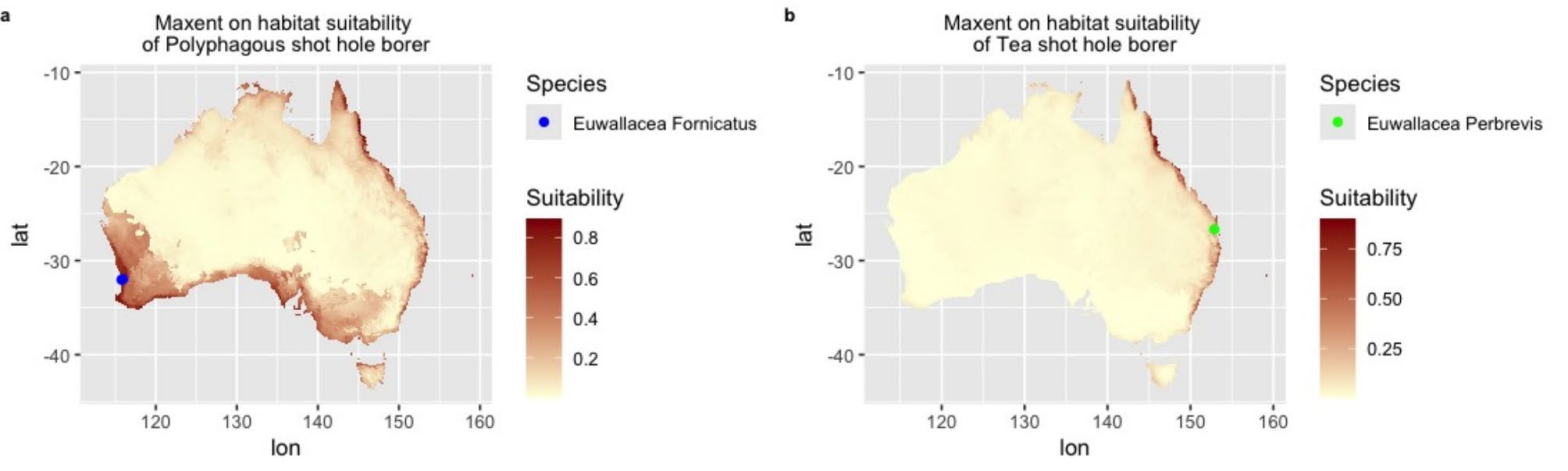
Predicted habitat suitability maps for *Euwallacea fornicatus* and *Euwallacea perbrevis* across Australia with Maxent model. The blue dot and green dot indicates occurrence records of *Euwallacea fornicatus* in Western Australia *and Euwallacea perbrevis* in Queensland respectively. Global bioclimate data were acquired from the WorldClim open database (https://worldclim.org). The Species Distribution Modelling of Maxent model was conducted using R Studio (Version 4.4.0) (http://www.rstudio.com/). Occurrence records of *Euwallacea fornicatus* and *Euwallacea perbrevis* in Australia were obtained from Global Biodiversity Information Facility (GBIF; https://www.gbif.org/).

The Maxent model predictions for habitat suitability for *E. fornicatus* and *E. perbrevis* (Fig. 4) generally align more closely with the environmental favourability outputs rather probability of presence. For *E. fornicatus*, the habitat suitability across Australia is consistent with the environmental favourability predicted by GLM and GAM models, though discrepancies are noted in Western Australia (WA) by BRT model. Meanwhile, habitat suitability for *E. perbrevis* across Australia are broadly consistent with the environmental favourability as indicated by GLM, GAM and BRT models, although Maxent model predicted lower suitability in northern Australia including northern part of Northern Territory (NT) and northern WA.

## Comparison of two species based on environmental favourability

Generally, *E. fornicatus* has a wider environmental favourability range in areas adjacent to the ocean and including all Australian states and territories (Fig. 2a, c, e ). Notably, extensive favourable areas for *E. fornicatus* are observed in Southeastern WA and northern Queensland (Qld). By comparison, the environmental favourability values of *E. perbrevis* (Fig. 3a, c, e ) generally revealed a narrower distribution with the highlighted areas in coastal regions of the eastern and northern states of Australia. Particularly, *E. perbrevis* shows high environmental favourability along the eastern coastline of Australia, including Qld, New South Wales (NSW), and Victoria (Vic).

**Fig. 5:**
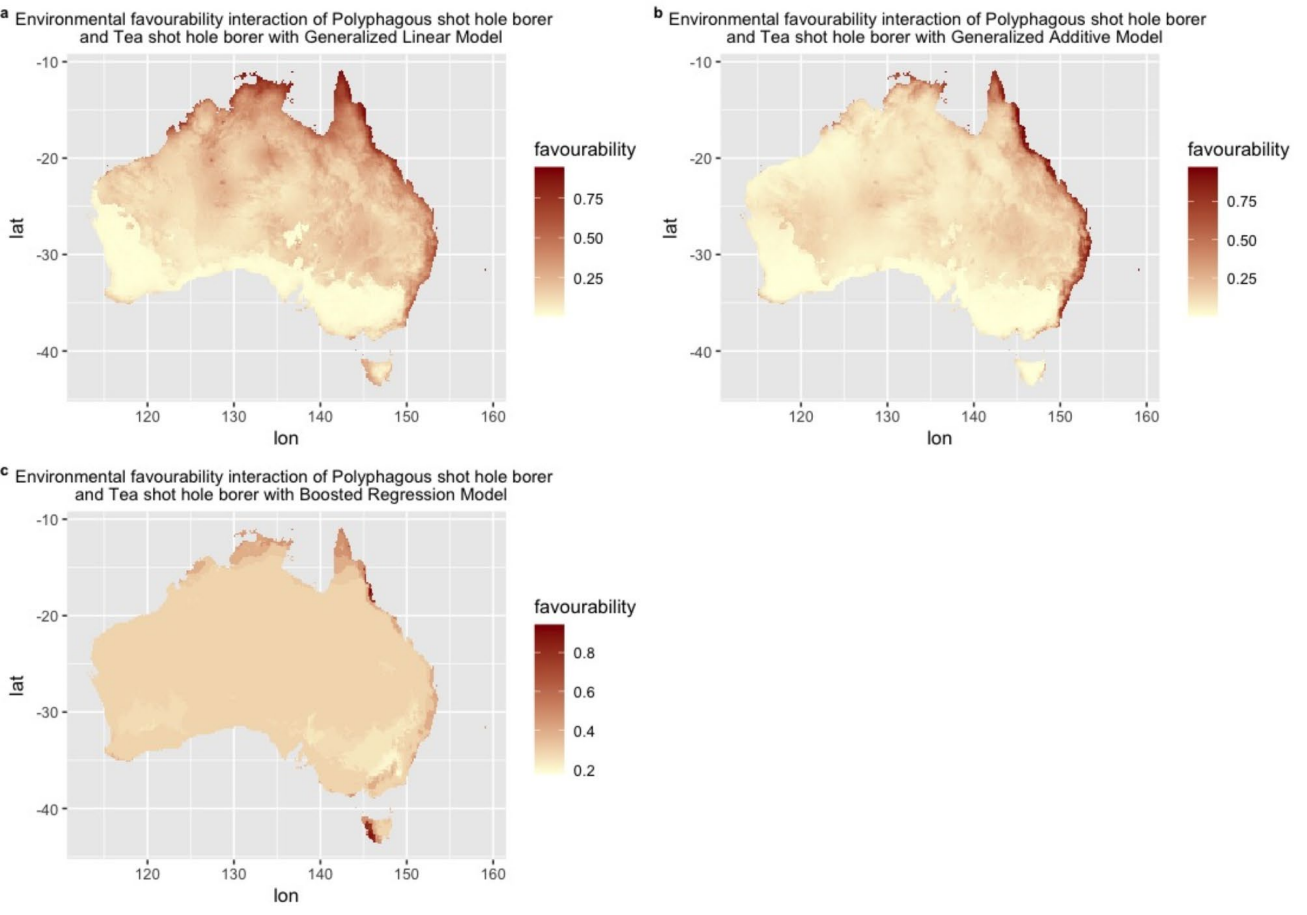
Illustration maps of environmental favourability intersection for *Euwallacea fornicatus* and *Euwallacea perbrevis* in Australia based on multiple Species Distribution Models: (**a**) Generalized Linear Model, (**b**) Generalized Additive Model, (**c**) Boosted Regression Model. Global bioclimate data were acquired from the WorldClim open database (https://worldclim.org). The Species Distribution Model, Favourability Function and Environmental Favourability Intersection was conducted using R Studio (Version 4.4.0) (http://www.rstudio.com/).

An intersection map was generated from the environmental favourability predictions for both *E. fornicatus* and *E. perbrevis* based on GLM, GAM and BRT model (Fig. 5). The maps are coloured from light to dark red representing the degree of intersection in the co-occurrence patterns of both species. The intersection maps reveals substantial overlap in environmental favourability for two species across eastern Australia, including Qld, NSW, Vic and NT.

Besides, the intersection map (Fig. 5) and the pair-wise similarity table (Appendix Table 1) reveals that the fuzzy similarity values calculated from favourability values are significantly higher than the binary similarity values derived from presence probability.

**Fig. 6:**
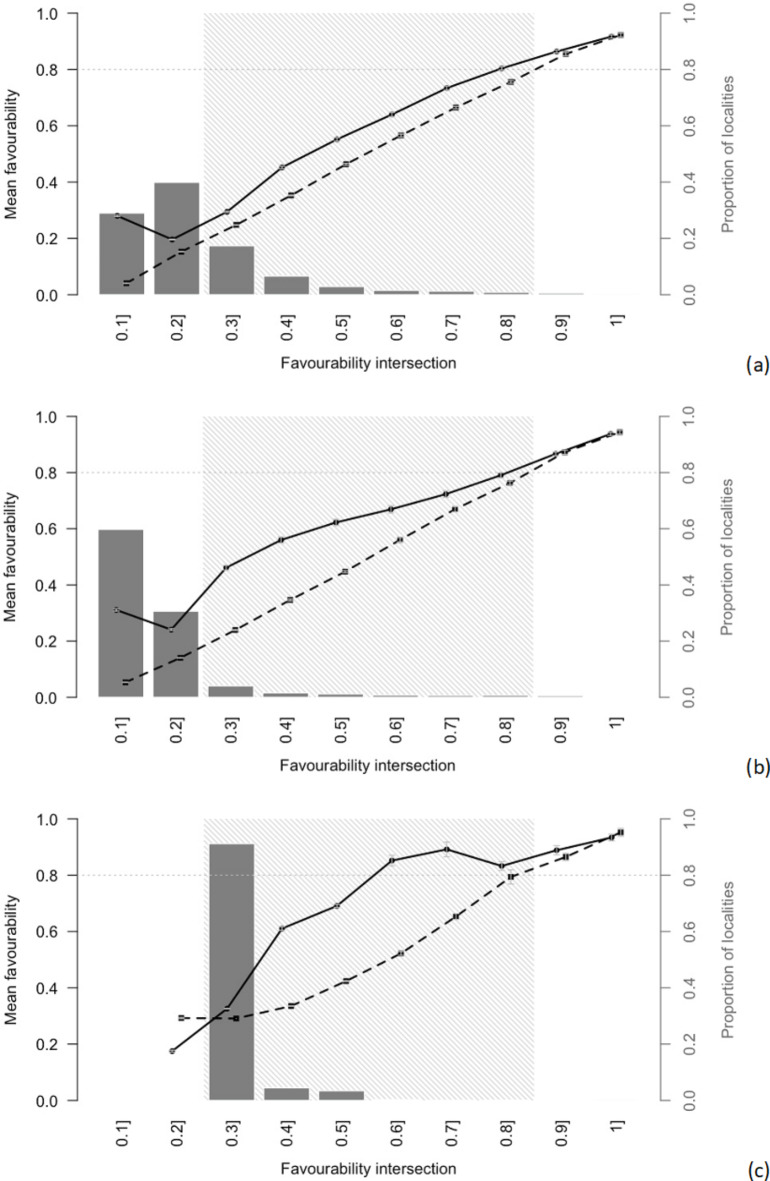
Plot of shared environmental favourability based on fuzzy overlap patterns inform biogeographical relationship and co-occurrence of two borer species *Euwallacea fornicatus* (continuous line) versus *Euwallacea perbrevis* (dashed line) with multiple Species Distribution Models: (**a**) Generalized Linear Model, (**b**) Generalized Additive Model, (**c**) Boosted Regression Model.

Combining the intersection maps of environmental favourability with the shared environmental favourability plot (Fig. 6) highlights the relationship between *E. fornicatus* and *E. perbrevis* in terms of environmental favourability overlap values along with the gradient defined by the FOvI gradient. The plot shows that the environmental favourability for *E. fornicatus* (continuous lines) is generally higher than *E. perbrevis* ( dashed lines) at each locality across all SDMs. The shared area with 0.2 < FOvI < 0.8 in (Fig. 6) illustrates intermediate environmental favourability for both *E. fornicatus* and *E. perbrevis*. In this shadowed interval, *E. fornicatus* showed higher environmental favourability values than *E. perbrevis*, with the FOvI between 0.2 and 0.6, while *E. perbrevis* attained higher environmental favourability values when FOvI exceeds 0.8. Outside the shadow, the left side non-shared area with FOvI < 0.2 indicated less favourability for *E. perbrevis*. The right side non-shared area with FOvI > 0.8 specified favourability for both *E. fornicatus* and *E. perbrevis* (Fig. 4).

### Model performance evolution

Both GAM and Maxent exhibited higher values across most metrics (Table 2). All models demonstrated robust discrimination capacity with high AUC values above 0.9, indicating excellent discrimination capacity^62^. However, GLM for *E. perbrevis* had a slightly lower AUC value of 0.882. Notably, Maxent achieved the highest AUC value 0.950 and 0.952 for *E. fornicatus* and *E. perbrevis* respectively. GAM for *E. perbrevis* showed the highest AUC value 0.956 indicating its superior discrimination capacity. All models exhibited TSS values greater than 0.6, with GAM achieving the highest TSS values of 0.755 for *E. fornicatus* and 0.748 for *E. perbrevis.* These high TSS values suggest that overall GAM provided the most accurate performance in predicting the presence and absence of the studied species. Conversely, BRT achieved the lowest overall performance with TSS value for both *E. fornicatus* and *E. perbrevis* respectively. GLM showed a higher TSS value of 0.752 for *E. perbrevis* but the lowest value of 0.6 for *E. fornicatus.* Maxent exhibited the highest CCR value of 0.966 and 0.964 for *E. fornicatus* and *E. perbrevis* respectively. Among GLM, GAM and BRT, BRT had a higher CCR value of 0.781 for *E. fornicatus*, while BRT had a higher CCR value of 0.915 for *E. perbrevis.* These high CCR values reflect the models strong accuracy and overall adequacy. In terms of Sensitivity and Specificity, GAM demonstrated the highest Sensitivity and moderate high Specificity, while Maxent exhibited the lowest Sensitivity but the highest Specificity compared to other SDMs (Table 2).

**Table 2:** Evaluation metrics for comparing the performance of four species distribution models for *Euwallacea fornicatus* and *Euwallacea Perbrevis.*

SDMs	Species metrics	AUC	TSS	CCR	Sensitivity	Specificity
GLM	*Euwallacea fornicatus*	0.882	0.600	0.756	0.844	0.756
	*Euwallacea perbrevis*	0.927	0.752	0.849	0.903	0.849
GAM	*Euwallacea fornicatus*	0.939	0.755	0.781	0.974	0.781
	*Euwallacea perbrevis*	0.956	0.748	0.861	0.887	0.861
BRT	*Euwallacea fornicatus*	0.907	0.605	0.774	0.831	0.774
	*Euwallacea perbrevis*	0.908	0.608	0.915	0.694	0.915
Maxent	*Euwallacea fornicatus*	0.950	0.628	0.966	0.662	0.966
	*Euwallacea perbrevis*	0.952	0.658	0.964	0.694	0.964

Based on the results of the HL test results (Table 3), BRT model exhibited extremely high Chi-square values, exceeding 10 for both *E. fornicatus* and *E. perbrevis.* Additionally, BRT recorded the highest RMSE and lowest P-value below 0.05, indicating poor model performance relative to other SDMs. In contrast, GAM demonstrated the lowest Chi-square values of 0.904 for *E. perbrevis* and 1.132 for *E. fornicatus*, as well as lowest RMSE and highest P-value of 0.997 and 0.998 for *E. fornicatus* and *E. perbrevis* respectively. These results suggest superior model performance for GAM. The Maxent model was not assessed using HL test as it provides output in terms of relative habitat suitability rather than direct probability of presence.

**Table 3:** Hosmer-Lemeshow test results for species distribution models of *Euwallacea fornicatus* and *Euwallacea Perbrevis* in Australia.

SDMs	Species	Chi.sq	*P*-value	RMSE
GLM	*Euwallacea fornicatus*	6.686	0.571	1.596
	*Euwallacea perbrevis*	5.487	0.704	1.084
GAM	*Euwallacea fornicatus*	1.132	0.997	1.038
	*Euwallacea perbrevis*	0.904	0.998	0.627
BRT	*Euwallacea fornicatus*	18.319	0.003	4.628
	*Euwallacea perbrevis*	25.951	0.000	9.597

## Discussion

Species Distribution Modelling (SDM) has been widely used for predicting potential distribution of species. However, the outputs of SDMs are mostly impacted by prevalence. This inevitable restriction makes comparison between different species or different study areas more difficult. This study introduced the rules of fuzzy logic to optimize SDM for predicting the environmental favourability of two borer species *E. fornicatus* and *E. perbrevis*, two borer clades that have been confused for many years. The results of this study demonstrate that fuzzy logic is capable of effectively optimizing SDM by comparing outputs across various regions and species to yield more informative outputs. This study employed environmental favourability function across three SDMs including Generalised Linear Model (GLM), Generalised Additive Modelling (GAM) and Boosted Regression Trees (BRT). These models were employed to assess and compare two recently classified complex species *E. fornicatus* and *E. perbrevis* based on their favourable habitats in Australia. Additionally, a Maxent model was introduced to provide a comparative analysis of the relative habitat suitability.

The comparative performance of the Species Distribution Models (SDMs) analysed in this study reveals significant insights into their relative effectiveness. Both GAM and Maxent outperformed GLM and BRT across most metrics. Specifically, GAM and Maxent achieved consistently high AUC values, indicating excellent discrimination capabilities. Meanwhile, GAM also displayed the lowest Chi-square values and RMSE, along with the highest P-values, suggesting it offers superior model performance. In contrast, BRT exhibited the lowest overall performance for both *E. fornicatus* and *E. perbrevis*, and its Chi-square values were extremely high, coupled with the highest RMSE and the lowest P-values. These metrics indicated that the prediction of BRT was less reliable compared to other models. Overall, GAM and Maxent showed superior performance in this research, while BTR demonstrated less efficacy across most metrics, indicating lower reliability in this context. Therefore, subsequent analyses primarily focused on the outputs of GAM, GLM and Maxent models.

Maxent predictions of habitat suitability with the Maxent model closely align with environmental favourability predictions with GAM and GLM, which validates the accuracy of both SDM predictions and Maxent’s performance. This verified that Maxent effectively models scenarios with presence and absence data^51^. However, the environmental favourability based on fuzzy logic provides the capacity to compare across different regions and species^2^. In other words, the predictions of Maxent represent relative habitat suitability for the studied species, indicating how suitable a habitat is compared to other locations within a given study area^51^. In contrast, the predictions of environmental favourability offer a direct measure of how favourable a specific environmental condition is for a species. The environmental favourability has a greater focus on interpreting the environmental conditions most conducive to the species. Consequently, while relative predictions of Maxent provide valuable insights within a specific context, they may be less intuitive for comparing and assessing absolute suitability across different study areas or species. In comparison, the environmental favourability predictions directly reflect the absolute favourability of different areas, which offers a more interpretable and comparable measure across various regions and species^2,69^.

Environmental *favourability* value is easily confused with the presence *probability* value. From this study, the apparent difference of presence *probability* and environmental *favourability* were mapped (Figs. 2 and 3). Notably, the environmental *favourability* values indicate a wide proportion of favourable habitats for both *E. fornicatus* and *E. perbrevis*, while the distribution of presence *probability* showed a much smaller number of suitable habitats for these invasive borers. Unlike conventional *probability*/*suitability* maps, our maps used environmental *favourability* values to indicate the degree of membership to each pixel along with more continuous variation across the Australian continent. Environmental *favourability* can indicate spatial variation more directly with a range of tendencies^9^ without being dominated by species *prevalence*^9^. To clarify, the higher environmental *favourability* values are not showing the *probability* of finding *E. fornicatus* and *E. perbrevis* in the coloured regions, rather it is showing the extent to which each species belongs to the fuzzy sets: unfavourable to favourable.

Furthermore, the fuzzy distribution similarity value (Appendix Table 1) between *E. fornicatus* and *E. perbrevis* are markedly higher than binary similarity values. This is because the similarity of species distribution is greater than the precise coincidence among their recorded occurrence points^7^. To be specific, the binary similarity output overlap is less because it is based on the conventional crisp value with defined boundaries, a given value that either belongs to a set (1) or set (0)^73^. Conversely, the fuzzy approach incorporates favourability values that offer a more nuanced representation of species. This approach yields higher pair-wise similarity values because the fuzzy set’s membership degree ranges from 0 to 1, allowing for more gradual transitions between favourable and unfavourable areas^5,73^. Consequently, the fuzzy environmental favourability maps provide more information that not only contains favourable or not-favourable areas, but also the transition areas that fall in between favourable or not-favourable, as illustrated in Figs. 2 and 3.

According to the predicted outputs (Figs. 2b, d and f and 3b, d and f), all Australian occurrence records for *E. fornicatus* and *E. perbrevis* fall within the predicted areas of environmental favourability, as indicated by all models, including GLM, GAM and BRT. This alignment is observable in the prediction maps (Figs. 2b, d and f and 3b, d and f). Despite this, the values of presence probabilities were generally low across all the three modelling algorithms, the GAM model predictions (Fig. 2c) align with occurrence records for *E. fornicatus* in WA, and the GLM model predictions (Fig. 3a) correspond with occurrence records for *E. perbrevis.* Additionally, the prediction outputs of the Maxent model align with occurrence records of both species, which indicates high suitability for *E. fornicatus* in WA and for *E. perbrevis* in Qld (Fig. 4).

The environmental favourability analysis based on various SDMs highlighted potential expansion regions for the two invasive borers *E. fornicatus* and *E. perbrevis* across Australia (Figs. 2 and 3). All the favourable habitats for *E. fornicatus* and *E. perbrevis* are around littoral areas. Specifically, the favourable habitat for *E. fornicatus* in WA (Fig. 2) aligns with recent records of *E. fornicatus*, detected in 2021 ^74^. Our analysis suggests considerable potential for expansion of *E. fornicatus* through WA as well as favourable conditions across other states and territories of Australia. This poses a potential threat to Qld and northern NSW where established populations of *E. perbrevis* are already present. Queensland Government has also identified the possibility of *E. fornicatus* establishment and spread in Qld^75^.

Conversely, *E. perbrevis* shows concentrated favourability along the eastern coastline of Australia (Fig. 3), including QLD and NSW, which corresponds with current distribution records. Favourable areas for *E. perbrevis* also extend into Victoria (Vic) and include a few predicted favourable areas in Tasmania (Tas). However, the probability of *E. perbrevis* establishing in other states and territories appears to be low, as indicated by the minimal environmental favourability predictions for WA. So far, there is no evidence of *E. perbrevis* presence in Australia, apart from Qld and NSW^75^. The reason might be that *E. perbrevis* finds the environment of other states to be less favourable, as our results show. Interestingly, *E. fornicatus* has recently arrived and is currently considered present but under eradication in WA, coinciding with the high environmental favourability shown for WA and Tas. However, all these states have a low environmental favourability for *E. perbrevis.* This illustrates the apparent difference in distribution patterns of the two borers, even though *E. fornicatus* has a similar biology and taxonomy to *E. perbrevis.*

The first detection of *E. fornicatus* was in the suburb of East Fremantle, Perth in August 2021 with symptoms of dieback and dead branches from a maple tree^74^. Frustratingly, the known range grew quickly in one year as surveillance expanded. It has now extended to over 80 suburbs and 25 councils across the Perth metropolitan area. It poses a serious threat to trees on private properties, in parks, along streets verges, in public open spaces and in reserves^76^, including ornamental species such as *Acer negundo* (Box Elder Maple) and *Ficus macrophylla* (Moreton Bay Fig), and as a result may seriously impact urban canopy cover. Most concerning is the detection of *E. fornicatus* on Rottnest Island^32^, which highlights its capacity for rapid and extensive spread facilitated by wind currents. Worthy of mention is that the changeable host range of *E. fornicatus*, which is likely influenced by different habitats and illustrated by *E. fornicatus*’s infestation on non-reported hosts such as mulberry and lime in WA^75^. For example, *E. perbrevis* was established in Qld for many years^77^ and it was reported to attack diseased macadamia trees^78^ rather than threatening avocado production in Qld. However, internationally the cases of *E. perbrevis* are regarded as a serious threat to avocado production in Israel and California^16^.

In addition, the mutualist Ambrosia Fusarium Clade (AFC) associated with *E. fornicatus* is another potential factor influencing the pest’s host range of the pest, as supported by detection of other *Fusarium* spp.in WA^75^. As is the case in Israel and California, *E. perbrevis* is associated with its mutualist fungus *Fusarium ambrosium*, which was a major problem to the avocado industry as the *Fusarium* species with mutualist fungus can cause disease in avocado^18,19^. Therefore, it can be suggested that the fungus associated with both *E. fornicatus* and *E. perbrevis* tend to result in a varying degrees of damage to different hosts depending on the region. Beyond the fungal *Fusarium euwallaceae*, which is mainly associated with *E. fornicatus*^18^, *E. fornicatus* may also associate with the fungal symbionts of *E. perbrevis* within AFC. If *E. fornicatus* has the chance to arrive and establish in Qld, this may cause unpredictable threats across various hosts.

Primarily, the potential threat of *E. fornicatus* and *E. perbrevis* to avocado and other hosts are both unpredictable and concerning. The avocado industry in Australia has experienced a rapid growth, coinciding with an increase in the size of avocado orchards^79^. Qld is the primary producer, with WA also increasingly contributing to Australian avocado production^79^. These important issues illustrate the uncertainty of the economic impact of the borer in Australia. As such, if *E. fornicatus* arrived in Qld, it may readily establish with a wider host range assisted by its new symbiotic relationship with alternative *Fusarium* species.

The possible pathway for spread of *E. fornicatus* mainly include carriers like wood, living plants, as well as timber machinery^75^. Since *Euwallacea* spp. are able to conceal themselves in the galleries within the woody host, the infestation can be hard to detect as the galleries are small and the pests are not very active^80^. Indeed, Qld has strict monitoring action on the borer and restrictions on transferring plant materials to avoid infested plants, wood and some machinery^28^. In fact, WA already has in place a containment program involving a group of restrictions for preventing the spread of another wood borer, *Hylotrupes bajulus* Linnaeus, European house borer from WA to other states and territories^81^.

*Euwallacea fornicatus* and *Euwallacea perbrevis* are known to co-exist in various regions around the world, which has led to their misidentification over many years. This study has provided a clear delineation of the distinct and shared distribution patterns of these two borers in Australia. To analyse the biogeographical relationships, the shared environmental favourability plot provides a summary view of the environmental favourability overlay between *E. fornicatus* and *E. perbrevis*. Based on the current presence data, no distinct spatial segregation is evident between *E. fornicatus* and *E. perbrevis*.

As indicated by the shared favourability gradients and fuzzy overlap patterns, *E. fornicatus* demonstrates a higher environmental favourability within Australia in comparison to *E. perbrevis.* Furthermore, the two borers exhibit a predominantly positive relationship, indicating a definite coexistence of the borers in terms of climate preference. In light of the positive environmental favourability for both *E. fornicatus* and *E. perbrevis*, potential biotic interactions could arise given their shared mutualist AFC, notwithstanding the minor differences in their respective susceptible hosts. This study identified the areas with favourableness to both borers, which can be considered as potential sympatric coexistence. The notion of ‘favourableness’^82^ gives an ideal species relative environmental fitnesses, determining whether the competing species may be able to coexist or not. The favourableness was not only used in the context of competing species, but also applied to the biogeographical relationship between positively related species^36,83^. Even though the two borers current have different distribution areas in Australia, their very similar biological characteristics and morphology make the similar potentially favourable habitats worthy of consideration. Thus, given the possible coexistence based on their positive relationship, as well as assistance from their possible shared mutualist AFC, we consider there is high risk of establishment across Australia.

## Conclusion

Based on the rules of fuzzy logic, the value of environmental favourability makes the predicted outputs comparable among multiple species with different prevalence. The results of this study provide a direct comparison of the biogeographical relationship between two closely related Australian invasive borers in the overall landscape. Considering the uncertainty surrounding susceptible hosts of *E. fornicatus* in new regions and the undescribed AFC in Australia, as well as the observed positive correlation in environmental favourability between *E. fornicatus* and *E. perbrevis*, it is crucial to note the potential risk posed by *E. fornicatus* to states and territories beyond Western Australia. The concept of environmental favourability demonstrates considerable potential in the realm of environmental geography, as it provides a comparative value that can be used between related species. It is a valuable tool in biogeography, enabling a comprehensive understanding of biogeographic relationships among related species in a given environment. A future study could encompass biological threat assessment of the pests with their susceptible hosts, as well as biological control and management of both *E. fornicatus* and *E. perbrevis* with a natural enemy. Applying fuzzy logic to SDM to optimize its application will yield more realistic outcomes for predicting species distribution patterns without the domination of prevalence. Environmental favourability deserves more practical and empirical application in species distribution management.

## Electronic supplementary material

Below is the link to the electronic supplementary material.

Supplementary Material 1

## Data Availability

All data generated or analysed during this study are included in the published article and its supplementary information files. Additionally, these datasets are available from the corresponding author upon reasonable request.
