# Supplementary material for: Assessment of fuzzy logic to enhance species distribution modelling of two cryptic wood boring beetle species in Australia
Source: Sci Rep. 2024 Nov 13;14:27871. doi: 10.1038/s41598-024-77533-0 (PMC11561313; doi:10.1038/s41598-024-77533-0)
Supplement: Supplementary file 1 — Supplementary Material 1 [file 41598_2024_77533_MOESM1_ESM.docx]

**Appendix**

| **Pair-wise similarity** | **Pair-wise binary similarity** | Euwallacea fornicatus | Euwallacea *perbrevis* |
| --- | --- | --- | --- |
| **GLM** | Euwallacea fornicatus | 1 | 0.053 |
|  | Euwallacea *perbrevis* | 0.053 | 1 |
|  | **Pair-wise fuzzy similarity** | Euwallacea fornicatus | Euwallacea *perbrevis* |
|  | Euwallacea fornicatus | 1 | 0.354 |
|  | Euwallacea *perbrevis* | 0.354 | 1 |
|  | | | |
|  | **Pair-wise binary similarity** | Euwallacea fornicatus | Euwallacea *perbrevis* |
| **GAM** | Euwallacea fornicatus | 1 | 0.051 |
|  | Euwallacea *perbrevis* | 0.051 | 1 |
|  | Pair-wise fuzzy similarity | Euwallacea fornicatus | Euwallacea *perbrevis* |
|  | Euwallacea fornicatus | 1 | 0.531 |
|  | Euwallacea *perbrevis* | 0.531 | 1 |
|  | | | |
|  | **Pair-wise binary similarity** | Euwallacea fornicatus | Euwallacea *perbrevis* |
| **BRT** | Euwallacea fornicatus | 1 | 0.053 |
|  | Euwallacea *perbrevis* | 0.053 | 1 |
|  | **Pair-wise fuzzy similarity** | Euwallacea fornicatus | Euwallacea *perbrevis* |
|  | Euwallacea fornicatus | 1 | 0.420 |
|  | Euwallacea *perbrevis* | 0.420 | 1 |

**Table 1.** Pair-wise binary similarity and fuzzy similarity of distributional relationship between Euwallacea fornicatus and *Euwallacea perbrevis* based on Jaccard index across multiple Species Distribution Models: (a) Generalized Linear Model, (b) Generalized Additive Model, (c) Boosted Regression Model.
